# Supplementary material for: Contribution of Hypoalbuminemia and Anemia to the Prognostic Value of Plasma p-Cresyl Sulfate and p-Cresyl Glucuronide for Cardiovascular Outcome in Chronic Kidney Disease
Source: J Pers Med. 2022 Jul 28;12(8):1239. doi: 10.3390/jpm12081239 (PMC9410048; doi:10.3390/jpm12081239)
Supplement: Supplementary file 1 [file jpm-12-01239-s001.zip › jpm-1778016-supplementary.pdf]

Journal of Personalized Medicine

MDPI

Supplementary Materials

Table S1. Regression model of factors associated with total pCS concentrations.

Factor	Beta Coefficient	95%CI	*p*
(Constant)		−0.048; 1.877	0.063
Hemoglobin (g/dL)	−0.102	−0.107; −0.010	0.019
Albumin (g/L)	0.062	−0.006; 0.042	0.145
eGFR (mL/min/1.73 m^2)	−0.478	−0.020; −0.015	<0.001

CI: confidence interval; eGFR: estimated glomerular filtration rate; pCS: p-cresyl sulfate.

Table S2. Regression model of factors associated with total pCG concentrations.

Factor	Beta Coefficient	95%CI	*p*
(Constant)		0.888; 2.874	<0.001
Hemoglobin (g/dL)	−0.146	−0.134; −0.033	0.001
Albumin (g/L)	−0.002	−0.025; 0.024	0.970
eGFR (mL/min/1.73 m^2)	−0.389	−0.017; −0.011	<0.001

CI: confidence interval; eGFR: estimated glomerular filtration rate; pCG: p-cresyl glucuronide.

Table S3. Regression model of factors associated with free pCG concentrations.

Factor	Beta coefficient	95%CI	*p*
(Constant)		1.239; 3.210	<0.001
Hemoglobin (g/dL)	−0.138	−0.129; −0.030	0.002
Albumin (g/L)	−0.030	−0.033; 0.016	0.481
eGFR (mL/min/1.73 m^2)	−0.410	−0.018; −0.012	<0.001

CI: confidence interval; eGFR: estimated glomerular filtration rate; pCG: p-cresyl glucuronide.

Table S4. Association between log(TpCS) and outcome.

Variable	HR	95% CI	*p*
Age (1 SD)	1.71	1.34; 2.18	<0.001
Sex (male = 1)	1.70	1.20; 2.42	0.003
Diabetes (yes = 1)	2.70	1.93; 3.77	<0.001
SBP (1 SD)	1.37	1.17; 1.61	<0.001
Log(TpCS) (1 SD)	1.38	1.14; 1.66	0.001
Albumin (1 SD)	0.61	0.51; 0.72	<0.001

Hazard ratio's (HR) for continuous variables are expressed per 1 standard deviation (SD) (age = 17 years, SBP = 20 mmHg, albumin = 3.46 g/L); CI: confidence interval; TpCS: total p-cresyl sulfate; SBP: systolic blood pressure.

Table S5. Association between log(TpCG) and outcome.

Variable	HR	95% CI	*p*
Age (1 SD)	1.82	1.43; 2.30	<0.001
Sex (male = 1)	1.88	1.32; 2.68	<0.001
Diabetes (yes = 1)	2.81	2.01; 3.92	<0.001
SBP (1 SD)	1.37	1.16; 1.61	<0.001
Log(TpCG) (1 SD)	1.33	1.11; 1.59	0.002
Albumin (1 SD)	0.62	0.52; 0.74	<0.001

Hazard ratio's (HR) for continuous variables are expressed per 1 standard deviation (SD) (age = 17 years, SBP = 20 mmHg, albumin = 3.46 g/L); CI: confidence interval; TpCG: total p-cresyl glucuronide; SBP: systolic blood pressure.

Table S6. Association between log(FpCG) and outcome.

Variable	HR	95% CI	*p*
Age (1 SD)	1.82	1.43; 2.31	<0.001
Sex (male = 1)	1.80	1.27; 2.57	0.001
Diabetes (yes = 1)	2.72	1.94; 3.81	<0.001
SBP (1 SD)	1.36	1.16; 1.61	<0.001
Log(FpCG) (1 SD)	1.30	1.09; 1.55	0.003
Albumin (1 SD)	0.62	0.52; 0.74	<0.001

Hazard ratio's (HR) for continuous variables are expressed per 1 standard deviation (SD) (age = 17 years, SBP = 20 mmHg, albumin = 3.46 g/L); CI: confidence interval; FpCG: free p-cresyl glucuronide; SBP: systolic blood pressure.
